# Effects of running a marathon on irisin concentration in men aged over 50

**DOI:** 10.1007/s12576-018-0619-3

**Published:** 2018-05-14

**Authors:** Paweł Jóźków, Dawid Koźlenia, Katarzyna Zawadzka, Marek Konefał, Paweł Chmura, Katarzyna Młynarska, Michał Kosowski, Marek Mędraś, Jan Chmura, Piotr Ponikowski, Jacek Daroszewski

**Affiliations:** 1 Department of Sports Medicine and Dietetics, Wroclaw University of Physical Education, Ul. I.J. Paderewskiego 35, 51-612 Wrocław, Poland; 2 Department of Team Sport Games, Wroclaw University of Physical Education, I.J. Paderewskiego 35, 51-612 Wrocław, Poland; 3 Department of Heart Diseases, Medical University, Wroclaw, L. Pasteur 4, 50-367 Wrocław, Poland; 4 Department of Endocrinology, Diabetes and Isotope Therapy, Wroclaw Medical University, L. Pasteur 4, 50-367 Wrocław, Poland

**Keywords:** Irisin, Endurance training, Running, Marathon, Men

## Abstract

Our aim was to verify whether running a marathon is associated with changes in irisin concentration in healthy, endurance-trained men. In an observational study, we assessed baseline biochemical and fitness parameters of 28 middle-aged runners (mean ± SD age, BMI, VO_2max_: 58 ± 8 years; 24.5 ± 3 kg/m^2^; 51.1 ± 1.7 ml/kg/min). We evaluated irisin before, immediately after, and 7 days after the marathon. Irisin concentration decreased from a baseline value of 639 ± 427 to 461 ± 255 ng/ml immediately after the marathon (*p* < 0.05). After 7 days, it was still significantly lower than before the race, at 432 ± 146 ng/ml (*p* < 0.05). We found no correlations between irisin concentration and the training history of the studied subjects. We conclude that a long-distance run may have a negative impact on irisin release in men. This effect was not correlated with the training history of runners.

## Introduction

Irisin is a relatively newly discovered peptide that affects metabolic processes. First described in 2012 [1], it has been shown to induce conversion of white adipose tissue (WAT) into brown adipose tissue (BAT) and thus affect energy metabolism [2].

Irisin provides a part of the transmembrane protein FNDC5 (fibronectin type III domain-containing protein 5) [3]. Transcription of FNDC5 mRNA is induced by PGC1α (peroxisome proliferator-activated receptor gamma coactivator 1-alpha), which affects cell metabolism. PGC1α increases the activity of mitochondria and enhances the turnover of glucose and triglycerides [4].

Original observations suggesting that irisin is stimulated by physical exercise sparked much interest in the scientific community. Irisin has been named an ‘exercise hormone’, meaning it conveys effects of exercise to the whole body. Initial research pointed to a possible role of irisin in the pathophysiology of insulin resistance, diabetes, and obesity [2]. Interestingly, irisin is supposed to have beneficial effects on energy homeostasis as well as anti-atherosclerotic, neuroprotective, and bone protective properties [5, 6].

Factors that might interfere with the above-mentioned effects of irisin include the type, duration and intensity of exercise [7] as well as the health status of an individual [8]. Contributions of age or sex on the irisin response have not been finally established [9]. Some [10] but not all [11] observations suggest that the release of this myokine may be more pronounced in the elderly. Interestingly, a recent meta-analysis showed that only fitness level is a predictor of the magnitude of post-exercise changes in irisin concentration [12].

Different from other approaches (often using a treadmill or cycle ergometer), we decided to investigate athletes taking part in a marathon run. A marathon is an example of an extreme endurance exercise [13], which has become increasingly popular in recent years. The run is associated with significant increases in proinflammatory interleukins, changes in blood protein/ion balance, fluctuations in hormone concentrations, and alterations in renal and liver function [14–17].

We wanted to verify whether irisin concentration changes after running a marathon. Our study aimed to assess the impact of a real-life, strenuous physical effort on irisin physiology in fit men. We collected a unique group of middle-aged runners homogenous in age, body mass index, health status, and fitness level. Such men pick up activities such as marathons due to their antiaging and rejuvenating effects [18]. This group fairly represents a growing part of the male population interested in a healthy lifestyle. The size of the group does not differ from the groups described in the literature [12]. To the best of our knowledge, this is the first investigation performed in a sample of male runners over the age of 50.

## Materials and methods

### Subjects and settings

We enrolled 28 healthy men (mean age, 58 years; range, 50–74) who trained to take part in the 32nd Wroclaw Marathon. This prestigious event has been organized continually since 1983. The competition was held in September at a temperature of 22 ± 2 degrees centigrade and a relative air humidity of 77 ± 13% (measurements were conducted by the Institute of Meteorology and Water Management—National Research Institute in Wroclaw). The start was set at 9:00 a.m. The number of participants exceeded 4000 (with 96% of them finishing the run).

In the preparation period, the studied men covered a weekly distance of 41 ± 19 km. This is a standard training volume for this age group and discipline [14, 19].

### Physiological assessment

Fourteen days before the marathon, each participant underwent an evaluation of VO_2max_ and HR_max_. The test was performed according to the Bruce protocol [20] on a Tmx Trackmaster treadmill (USA). For the first 3 min, the enrollees ran at a speed of 2.7 km/h, with a 10° incline. Then, the loads were changed according to the protocol. Heart rate was evaluated with a sport-tester M400 (Polar, Finland). Oxygen uptake was measured with an Ergostick machine (Reynolds Medical, USA).

### Biochemical methods

The participants underwent laboratory evaluations 2 weeks before the race (on the day VO_2max_ and HR_max_ were assessed). Blood samples were acquired from the basilic vein into tubes pre-loaded with ethylenediaminetetraacetic acid (EDTA) after at least 9 h of fasting. Whole blood samples were centrifuged at 4 °C for 15 min at 3000 rpm, and then the separated plasma was stored at − 80 °C until subsequent analysis. The laboratory was equipped with Konelab 60 analyzer (Thermo Scientific, Finland). The following methods were used to evaluate: glucose—a colorimetric method with GOD-POD oxidase; cholesterol—a colorimetric method with cholesterol esterase/cholesterol oxidase; triglycerides—a colorimetric method with lipase/GPO-PAP without correction; HDL cholesterol—a direct method with enzymes modified with polyethylene glycol (PEG-Kyowa, Medex, Japan). LDL was calculated using the Friedewald’s formula: LDL = total cholesterol − triglycerides/5 (mg/dl). Insulin was determined with ELISA kit using Liaison XL analyzer (DiaSorin, Italy). HOMA-IR was counted using the following equation: fasting insulin (mU/ml) × fasting glucose (mmol/l)/22.5. TSH, fT3, and fT4 were measured with the electrochemiluminescence method with use of Cobas e411 analyzer (Roche Diagnostics, Switzerland). The characteristics of the group are presented in Table 1.

**Table 1:** The anthropometric, sport, and biochemical characteristics of the study group

Parameter	Mean ± SD
Age (years)	58 ± 8
Height (cm)	174 ± 7
Weight (kg)	75.5 ± 11
BMI (kg/m^2^)	24.5 ± 3
Kilometers run per week (km)	40 ± 20
Years of training	9 ± 11
VO_2max_ (ml/kg/min)	51.1 ± 1.7
Maximal heart rate	170 ± 2
Fasting glucose (mg/dl)	90 ± 6
Insulin (µIU/ml)	6.54 ± 3.58
HOMA-IR	1.49 ± 0.9
Total cholesterol (mg/dl)	214 ± 25
LDL (mg/dl)	129 ± 28
HDL (mg/dl)	66 ± 20
TG (mg/dl)	100 ± 33
TSH (µIU/l)	2.09 ± 1.1
fT3 (pg/ml)	3.07 ± 0.29
fT4 (pg/ml)	1.22 ± 0.14

Irisin concentration was assayed from serum. Blood samples were collected before, immediately after the run, and 7 days after the marathon. They were centrifuged (after every collection), and the separated sera were placed in test tubes and frozen at − 85 °C. Irisin concentration was measured with use of an enzyme immunoassay kit ELISA (Biovendor, Brno, Czech Republic). Interassay CV = 6.91%, intraassay CV = 9.07%. The assay limit of detection was 0.001 μg/ml.

Concentrations of irisin and other biochemical parameters were recalculated with adjustments for exercise-induced fluctuations of plasma volume [21].

### Statistical analysis

Statistical analysis was performed using Statistica v.12 software. Statistical significance was set at *p* < 0.05. The Shapiro–Wilk’s test was used to verify whether the variables had normal distributions. The basic characteristics of the groups were analyzed using descriptive statistics and tests of differences between variables (dependent samples). If there were more than two variables that were measured in the same sample, then we would customarily use repeated-measures ANOVA. Some dependent variables did not have normal distributions, so Friedman’s analysis of variance and post hoc tests were used. Spearman’s correlation was used to verify the significance of correlations between variables.

## Results

The mean run time (± SD) was 04 h:16 min: 02 s ± 0 h: 31 min: 23 s. We found that the mean (± SD) serum concentration of irisin in the study participants before the race was 639 ± 426 ng/ml.

Immediately after the run, the mean concentration of irisin decreased by 30% to 449 ± 256 ng/ml (*p* < 0.05). Evaluations performed 7 days after the event showed that the irisin concentration was still low, at 432 ± 146 ng/ml. This value was 32% lower than at baseline (*p* < 0.05). The mean irisin concentration in samples acquired immediately after running and 7 days after the race did not differ significantly (Table 2).

**Table 2:** Post hoc tests evaluating the differences between irisin concentrations at the following studied time points: before (V1), immediately after (V2), and 7 days after the race (V3)

	Irisin V1 (ng/ml)	Irisin V2 (ng/ml)	Irisin V3 (ng/ml)
Irisin V1 (ng/ml)	–	1	0.71
Irisin V2 (ng/ml)	1	–	0.28
Irisin V3 (ng/ml)	0.71	0.28	–

The absolute differences between the average ranks were significant above 0.66 (*p* < 0.05)

We did not find any correlations between the mean irisin concentrations evaluated at the three time points and the duration of training (years) or the distance covered weekly by the studied athletes (Table 3).

**Table 3:** Correlations between the independent variables and irisin concentration

Spearman’s correlation	*p* value
Irisin V1 (ng/ml) and km/week	0.58
Irisin V1 (ng/ml) and training (years)	0.90
Irisin V2 (ng/ml) and km/week	0.65
Irisin V2 (ng/ml) and training (years)	0.56
Irisin V3 (ng/ml) and km/week	0.59
Irisin V3 (ng/ml) and training (years)	0.49

There were no correlations between other studied parameters (glucose, insulin, HOMA-IR, total cholesterol, HDL, LDL, triglycerides, TSH, fT4, fT3) and changes of the mean irisin concentration either.

## Discussion

In our study, running a marathon resulted in a decrease in serum irisin concentrations in fit, lean, middle-aged men. This effect was observed immediately after the run and 7 days after the race as well.

Irisin is an adipo-myokine involved in muscle-adipose tissue cross-talk [2]. The paracrine and endocrine effects of irisin comprise enhancements of glucose metabolism and increased oxygen consumption. Irisin is able to convert white adipose tissue into brown adipose tissue. It increases thermogenesis via activation of an uncoupling protein 1 (UCP1) [1]. We must admit that there are voices questioning the physiological role of irisin in humans as well [22].

Since 2012, when Bostrom et al. first described irisin, many research groups have analyzed the relationships between exercise and irisin release. The presence of such associations and the influence of different types, intensities, and durations of physical exercise on irisin production have been a matter of ongoing debate [7, 11, 12, 23–26].

Several studies have not shown any association between physical activity and irisin concentration; e.g., Pekkala et al. investigated the effects of exercise on irisin levels after a single bout of low-intensity aerobic training, a single resistance exercise, and 21 weeks of endurance or combined endurance/resistance training. In none of these programs did irisin concentration change [11]. Similar observations were reported by Hecksteden et al., who found no change in irisin levels in subjects undergoing 26 weeks of either endurance or strength training [26].

However, other reports showed associations between exercise and blood irisin levels. In many of these studies, the irisin response was dependent on the type and intensity of exercise [7, 23, 27]. In healthy, lean subjects, irisin concentration changed after a bout of high rather than lower intensity exercise [7, 23, 28].

Although observational studies tend to report on increases in irisin after chronic exercise, in controlled trials with randomization, this effect is not observed. One cannot exclude that high loads of exercise can reduce the release of irisin. For example, a 3-month training intervention in middle-aged men led to decreased irisin [29], and a 9-month training of elite-level tennis players was associated with decreased irisin concentration at the middle and at the end of the season [30].

Apart from exercise, other factors may affect irisin release in humans as well. Irisin is released in a day–night rhythm with a peak concentration observed in the morning [9]. Circadian variation of physiological responses to stress and exercise may play a role here [31]. Irisin production is also affected by the season of the year [8]. It seems rational that basal concentrations of irisin decrease with advancing age [10, 23]. Whether irisin response to exercise changes with advancing age has not been established. Difficulties and pitfalls encountered during laboratory measurements of irisin have been recently discussed extensively [32] and are far from being solved.

In light of the above-mentioned uncertainties, our results add some new information to the physiology of irisin in response to a popular exercise, the marathon run. A marathon is an example of a strenuous endurance effort [33]. The run is associated with increases in the cytokines IL-1, IL-6, IL-8, IL-10, and TNF-α [15]. The concentrations of total protein, albumin, calcium, phosphorus, and myoglobin increase, as does the anion gap [16]. Directly after a run, cortisol, epinephrine, and growth hormone concentrations are elevated, while insulin concentration is decreased. The levels of thyroid hormones, androstenedione, dehydroepiandrosterone, and estradiol rise, while testosterone level is reduced [17]. There have been observed elevations of creatinine, blood urea nitrogen, uric acid, creatine kinase, and lactate dehydrogenase [13]. Hepatic metabolism is enhanced, and the activity of enzymes such as lactate dehydrogenase, aspartate aminotransferase, alanine transaminase, g-glutamyltranspeptidase is increased. The concentration of bilirubin is higher after a run than before it [13, 16].

In contrast to Anastasilakis et al., who showed an increase in irisin after 30 min of outdoor running in young subjects (aged 20 ± 1) [9], we found that the irisin concentration decreased after running a marathon by approximately 30%. A similar decrease in irisin was shown by Belviranli et al. [34]. In the latter report, a high-intensity interval training was associated with reduced blood irisin levels (immediately after exercise) in elite kickboxers and sedentary individuals. The training sessions consisted of four rounds of 30-s maximal intensity cycling interspersed with 4 min of rest. Irisin concentration in kickboxers decreased by 37% and in sedentary subjects, by 16%. To a somewhat similar extent, Norheim et al. reported that 12 weeks of combined endurance and strength training reduced irisin concentration (of note, the acute irisin response was opposite) [29]. Interestingly, authors from Japan also found that exercise, this time of low-intensity, was associated with reduced irisin concentrations in six young, sedentary men [27]. What seems important is that in our study, irisin concentration after 7 days was still lower than that of baseline (by 32%). In the above-mentioned report by Belviranli et al., there was an observed tendency toward an increase of the myokine, which after 6 h, however, was still lower than that of baseline (by 17% in kickboxers and by 13% in sedentary controls) [34]. Other authors have reported that in young athletes, an effect of exercise on irisin concentration disappeared after 24 h [35]. Few protocols have used blood sampling days after a bout of exercise. We could attribute longer suppression of irisin to relatively older age and diminished post-exercise recovery abilities of our study subjects.

A factor that may influence irisin physiology is training status/fitness level [36]; e.g., athletes studied by Belviranli et al. had higher baseline irisin levels than age-matched sedentary controls (by 28%) [34]. The mean irisin concentration in our runners seemed to be higher than, e.g., in untrained elderly volunteers from the study by Miyamoto et al. [10]. Keeping in mind differences in methodology, type of physical activity, intensity of exercise, time to restoration, nutrition, etc., which make such comparisons hardly justified, we also acknowledge the results presented by Huh et al. who did not see any relation between changes of irisin and age/fitness [23]. At the same time, we have to note that the results of a recent meta-analysis associate the magnitude of post-exercise irisin response solely to the fitness level of studied subjects [12]. In our subjects, we did not observe any correlation between irisin variability and the training history. A possible explanation for this is the fact that sports experience (and thus athletic level) of our volunteers was relatively similar.

Irisin production and release is modified by a subtle interplay between hormonal and metabolic factors. Keeping in mind a possible role of thyroid hormones [37], we found that they were not related to marathon-induced changes of irisin concentration in our study subjects. It is also known that irisin exerts effects in glucose homeostasis [38]. We did not find any associations between fluctuations of irisin and changes of glucose, insulin or HOMA-IR in the three time points of our investigation either. Although acute or chronic fatigue may play a role here [39], we are still unable to determine it precisely. We may only hypothesize that a marathon is one of the most strenuous physical efforts undertaken by humans.

The effects of irisin in humans are not fully understood. To our knowledge, our study is the first to investigate associations between running a marathon and irisin concentration. As there are tens of thousands who run marathons and even more who train to participate in such events (for months or years), it seems prudent to explore relationships between this specific exercise and the physiology of this interesting myokine. We hope that understanding the regulation of the molecule in individuals of specific fitness levels and under different loads may translate into new therapeutic approaches in the future.

Among the strengths of the present investigation is the enrollment of participants aged over 50. As populations of developed countries age, the number of older runners rapidly grows. The fact that the studied group was homogenous as to ethnicity, health, previous training experience, and fitness level further enforces the presented findings. In contrast to many other studies, we evaluated the fitness level of all the volunteers and assessed their VO_2max_. In our calculations, we used the formula described by Dill to exclude possible bias arising from dehydration during physical effort [21]. Our subjects were lean; thus we avoided a bias introduced by excessive fat mass. Moreover, the participants did not present any co-morbidity, which makes it easier to draw conclusions about the physiology of irisin metabolism. We used a reliable laboratory method of irisin evaluation that has been previously applied in our setting [40, 41].

Among the shortcomings, we have to include a relatively small number of enrolled subjects. On the other hand, previously described samples were usually smaller than ours. The meta-analysis by Fox et al. comprised a total of 241 participants only [12]. Although caloric intake and diet were not evaluated in our project, they are thought to not interfere with irisin fluctuations [9].

## Conclusions

Running a marathon results in decreased concentration of irisin in endurance-trained, lean, healthy men aged over 50. This effect persists for at least a week. Changes in irisin concentration seem to not be dependent on the past training history of athletes.
